# Society Meetings

**Published:** 1907-07

**Authors:** 


### SOCIETY MEETINGS.

---

The Medical Society of the County of Erie, held its regular June meeting at the rooms of the Society of Natural Sciences, Buffalo library building, on Monday, June 17, 1907, at 4 P.M. Dr. A. H. Briggs is the president and Dr. F. C. Gram is the secretary.

---

The Buffalo Academy of Medicine held meetings in May and June as follows:

*Section on Obstetrics and Gynecology.*—Thursday, May 30, at 8:30 P.M. Program: Fibroid tumors of the uterus in pregnancy, labor and puerperal state, Matthew D. Mann, Election of officers of the section.

*Annual Meeting* of the Academy was held Tuesday, June 11, at 9 P.M. Election of officers from 8 to 9 P.M. Meeting of the council at 8:30 P.M. Program: President's Address, Charles S. Jewett.

The Sixth International Dermatological Congress will be held at New York, beginning September 9, 1907, and continuing for one week, under the presidency of Dr. James C. White, of Boston. Dr. Grover W. Wende, of Buffalo, is a member of the organisation committee and Dr. John A. Fordyce, 80 West 40th street, New York, is the secretary-general.

---

The Eleventh Antialcoholic Congress will be held at Stockholm, July 28, 1907. The Swedish government through its state and educational departments has taken full control of the congress, and formally invited every country in Europe, including the United States and Canada, to send representatives and eminent persons interested and acquainted with the subject. The state department at Washington has appointed as delegates for the government, Surgeon-General O'Reilly, U. S. Army and Medical Inspector Beyer of the Navy, Drs. T. D. Crothers of Hartford, Conn., and T. A. McNicholl of New York, to represent the medical profession and Mr. B. A. Hockhart of Hartford, to represent the temperance organisations and the Swedish people of this country.

---

The American Association of Obstetricians and Gynecologists will hold its twentieth annual meeting at Detroit, Tuesday, Wednesday, and Thursday, September 17, 18 and 19, 1907, under the presidency of Dr. Robert Tuttle Morris, of New York. Drs. J. H. Carstens, H. W. Longyear and W. B. Manton, all of Detroit, compose the committee of arrangements. An interesting program is in preparation.

